# Supplementary material for: Comparison of Lateral Flow Immunochromatography and Phenotypic Assays to PCR for the Detection of Carbapenemase-Producing Gram-Negative Bacteria, a Multicenter Experience in Mexico
Source: Antibiotics (Basel). 2023 Jan 6;12(1):96. doi: 10.3390/antibiotics12010096 (PMC9855030; doi:10.3390/antibiotics12010096)
Supplement: Supplementary file 1 [file antibiotics-12-00096-s001.zip › antibiotics-2103048-supplementary.pdf]

Comparison of lateral flow immunochromatography and phenotypic assays to PCR for the detection of carbapenemase-producing Gram-negative bacteria, a multicenter experience in Mexico.

Table S1. Oligonucleotide sequence for carbapenemases genes

Gene	Oligonucleotide sequence	Amplicon size (bp)
***bla**$_{NDM}$*	F: 5´- GGCGGAATGGCTCATCACGA-3´<br>R: 5´- CGCAACACAGCCTGACTTTC-3´	635
***bla**$_{IMP}$*	F: 5´- GTGATGCGTCYCCAAYTTCACT-3´<br>R: 5´- GGAATAGAGTGGCTTAATTCT-3´	435
***bla**$_{VIM}$*	F: 5´- AGTGGTGAGTATCCGACAG-3´<br>R: 5´- ATGAAAGTGCGTGGAGAC-3´	485
***bla**$_{KPC}$*	F: 5´- TTTTCAGAGCCTTACTGCCC-3´<br>R: 5´- ATGTCACTGTATCGCCGTGT-3´	798
***bla**$_{OXA\text{-}48\ like}$*	F: 5´- CCAAGCATTTTTACCCGCATCKAC-3´<br>R: 5´- GYTTGACCATACGCTGRCTGC-3´	438

Table S2. The proportion of genus and specie of the *Enterobacterales*.

K. pneumoniae	*E. coli*	*K. oxytoca*	*P. mirabilis*	*E. cloacae*	*E. ludwigii*	*M. morganii*	*C. freundi*	**Total**
25 (43%)	18 (31%)	4 (7%)	4 (7%)	3 (5%)	2 (3.4%)	1 (1.8%)	1 (1.8%)	58

Table S3. Complete description of Enterobacterales and P. aeruginosa collected.

ID Number	Hospital	Microbiology	Isolation Site	ETP	DOR	IMR	MEM	Genomic sequencing
EB2024	INR	*K. oxytoca*	Bone	2	4	4	2	KPC-2
EB2572	INR	*E. cloacae*	Blood	64	32	16	32	NDM-1
EB2637	INR	*E. coli*	Quantitative burn wound biopsy	1	2	2	2	OXA-232
EB2841	INR	*K. oxytoca*	Quantitative burn wound biopsy	1	2	2	2	OXA-232
EB2896	INR	*K. pneumoniae*	Quantitative burn wound biopsy	1	2	2	2	OXA-232
EB2988	INR	*K. pneumoniae*	Quantitative burn wound biopsy	2	2	1	1	OXA-232
EB2990	INR	*C. freundii*	Quantitative burn wound biopsy	1	1	1	1	OXA-232
EB2991	INR	*K. pneumoniae*	Quantitative burn wound biopsy	1	1	1	1	OXA-232
EB2993	INR	*K. pneumoniae*	Quantitative burn wound biopsy	1	1	1	1	OXA-232
EB3034	INR	*K. pneumoniae*	Bone	32	16	32	12	OXA-232
EB3081	INR	*K. pneumoniae*	Urine	32	16	32	12	OXA-48
EB3343	INR	*E. coli*	Urine	16	8	4	2	No carbapenemases detected
EB3349	INR	*K. pneumoniae*	Blood	64	64	32	64	NDM-1
EB3487	INR	*E. coli*	Blood	32	32	16	16	NDM-1/KPC-2
EB3627	INR	*K. pneumoniae*	Quantitative burn wound biopsy	4	2	2	2	OXA-232
EB3789	INR	*K. pneumoniae*	Blood	16	8	19	8	KPC-2
EB3806	INR	*K. pneumoniae*	Bone	4	2	2	1	OXA-232
EB3828	INR	*K. pneumoniae*	Bone	4	1	2	2	OXA-181
EB3877	INR	*E. coli*	Urine	1	1	1	1	OXA-48
EB3920	INR	*E. ludwigii*	Bone	8	8	8	8	NDM-1
EB4132	INR	*E. ludwigii*	Quantitative burn wound biopsy	8	16	8	16	NDM-1

EB4161	INR	*K. pneumoniae*	Urine	32	32	32	32	VIM-2
EB4286	INR	*M. morganii*	Bone	0.5	0.5	2	1	VIM-67
EB4328	GG	*K. pneumoniae*	Respiratory sample	16	8	4	2	No carbapenemases detected
EB4329	GG	*K. pneumoniae*	Respiratory sample	16	16	8	8	NDM-1
EB4330	GG	*K. pneumoniae*	Respiratory sample	16	8	8	4	NDM-1
EB4332	GG	*K. pneumoniae*	Bone	16	8	8	16	NDM-1
EB4335	GG	*E. coli*	Urine	8	2	4	1	KPC-3
EB4338	GG	*K. pneumoniae*	Blood	16	2	16	2	KPC-2
EB4339	GG	*K. pneumoniae*	Blood	16	2	16	1	KPC-2
EB4340	GG	*K. pneumoniae*	Urine	16	4	16	2	KPC-2
EB4350	HCG	*K. pneumoniae*	Blood	32	64	8	16	NDM-1
EB4354	HCG	*K. pneumoniae*	Blood	64	64	32	32	NDM-1
EB4360	HCG	*P. mirabilis*	Respiratory sample	32	64	64	32	NDM-1
EB4361	HCG	*P. mirabilis*	Respiratory sample	32	64	64	32	NDM-1
EB4371	HCG	*K. pneumoniae*	Urine	16	64	8	16	NDM-1
EB4375	HCG	*K. pneumoniae*	Blood	64	64	64	64	NDM-1
EB4376	HCG	*K. pneumoniae*	Urine	64	64	32	32	NDM-1/KPC-2
EB4390	HCG	*E. cloacae*	Soft tissue	32	32	8	16	NDM-1
EB4393	HCG	*E. coli*	Soft tissue	64	64	8	16	NDM-1
EB4394	HCG	*E. coli*	Urine	16	4	1	2	No carbapenemases detected
EB4396	HCG	*E. coli*	Urine	64	4	4	4	No carbapenemases detected
EB4399	HCG	*P. mirabilis*	Urine	64	64	32	32	NDM-1
EB4400	HCG	*P. mirabilis*	Soft tissue	16	64	32	32	VIM-2
EB4403	INCAN	*E. cloacae*	Biopsies	16	1	0.25	0.125	No carbapenemases detected

EB4404	INCAN	*E. coli*	Blood	8	4	0.062	0.125	No carbapenemases detected
EB4407	INCAN	*E. coli*	Respiratory sample	32	4	2	2	OXA-48
EB4409	INCAN	*K. oxytoca*	Urine	4	2	1	0.25	OXA-232
EB4411	INCAN	*K. oxytoca*	Urine	4	4	4	1	KPC-2
EB4412	INCAN	*K. pneumoniae*	Urine	4	8	8	1	KPC-2
EB4416	INCAN	*E. coli*	Urine	8	2	2	2	KPC-2
EB4426	INCAN	*E. coli*	Urine	1	0.25	1	0.125	OXA-181
EB4428	INCAN	*E. coli*	Urine	16	32	8	8	NDM-5
EB4430	INCAN	*E. coli*	Urine	1	0.125	0.25	0.125	No carbapenemases detected
EB4433	INCAN	*E. coli*	Bone	16	1	8	1	OXA-232
EB4435	INCAN	*E. coli*	Blood	2	1	1	0.25	OXA-232
EB4438	INCAN	*E. coli*	Urine	4	1	1	1	KPC-82
EB4440	INCAN	*E. coli*	Urine	1	0.125	0.125	0.062	OXA-232
P650	INR	*P. aeruginosa*	Quantitative burn wound biopsy	-	64	64	64	VIM-2
P661	INR	*P. aeruginosa*	Urine	-	64	64	64	IMP-75/NDM-1
P704	INR	*P. aeruginosa*	Urine	-	64	64	64	IMP-75
P766	INR	*P. aeruginosa*	Quantitative burn wound biopsy	-	64	64	64	VIM-2
P771	INR	*P. aeruginosa*	Quantitative burn wound biopsy	-	64	64	64	VIM-2
P773	INR	*P. aeruginosa*	Urine	-	64	64	64	VIM-2
P849	INR	*P. aeruginosa*	Bone	-	64	64	64	IMP-75
P850	INR	*P. aeruginosa*	Urine	-	64	64	64	IMP-75
P876	INR	*P. aeruginosa*	Urine	-	32	32	32	IMP-62
P921	INR	*P. aeruginosa*	Quantitative burn wound biopsy	-	64	64	64	IMP-15
P951	INR	*P. aeruginosa*	Urine	-	64	64	64	IMP-75
P973	INR	*P. aeruginosa*	Urine	-	16	64	64	VIM-2
P1017	INR	*P. aeruginosa*	Blood	-	64	64	32	VIM-2
P1028	INR	*P. aeruginosa*	Urine	-	64	64	64	VIM-2
P1044	INR	*P. aeruginosa*	Blood	-	32	64	64	VIM-2
P1157	INR	*P. aeruginosa*	Quantitative burn wound biopsy	-	32	64	64	VIM-2

P1870	INR	*P. aeruginosa*	Blood	-	16	16	16	IMP-62
P1875	INR	*P. aeruginosa*	Urine	-	32	8	32	NDM-1
P1885	INR	*P. aeruginosa*	Respiratory sample	-	16	16	16	No carbapenemases detected
P1886	INR	*P. aeruginosa*	Urine	-	32	32	32	No carbapenemases detected
P1897	INR	*P. aeruginosa*	Blood	-	32	16	64	IMP-62
P1910	INR	*P. aeruginosa*	Respiratory sample	-	32	32	32	VIM-2
P1921	INR	*P. aeruginosa*	Bone	-	8	16	16	No carbapenemases detected
P1948	GG	*P. aeruginosa*	Blood	-	16	64	64	VIM-2
P1949	GG	*P. aeruginosa*	Urine	-	16	64	64	VIM-2
P1928	GG	*P. aeruginosa*	Pleural fluid	-	8	8	16	VIM-2

INR: Instituto Nacional de Rehabilitación, HCG: Hospital Civil de Guadalajara Fray Antonio Alcalde, INCAN: Instituto Nacional de Cancerologia, GC: Hospital General Dr. Manuel Gea Gonzalez.
ETP: Ertapenem MICs, DOR: Doripenem MICs, IMR: imipenem MICs, MEM: Meropenem MICs
